# Effect of Co-administration of Bumetanide and Phenobarbital on Seizure Attacks in Temporal Lobe Epilepsy

**DOI:** 10.32598/bcn.9.6.408

**Published:** 2018-11-01

**Authors:** Reza Rahmanzadeh, Soraya Mehrabi, Mahmood Barati, Milad Ahmadi, Fereshteh Golab, Sareh Kazmi, Mohammad Taghi Joghataei, Morteza Seifi, Mazaher Gholipourmalekabadi

**Affiliations:** 1. Cellular and Molecular Research Center, Iran University of Medical Sciences, Tehran, Iran.; 2. Department of Biotechnology, School of Allied Medicine, Iran University of Medical Science, Tehran, Iran.; 3. Shefa Neuroscience Research Center, Tehran, Iran.; 4. Department of Anatomy, School of Medicine, Iran University of Medical Sciences, Tehran, Iran.; 5. Department of Medical Genetics, Faculty of Medicine and Dentistry, University of Alberta, Edmonton, Alberta, Canada.; 6. Department of Tissue Engineering & Regenerative Medicine, School of Advanced Technologies in Medicine, Iran University of Medical Sciences, Tehran, Iran.

**Keywords:** Bumetanide, KCC2, NKCC1, Phenobarbital, Temporal lobe epilepsy

## Abstract

**Introduction:**

The resistance of temporal lobe epilepsy to classic drugs is thought to be due to disruption in the excitation/inhibition of this pathway. Two chloride transporters, NKCC1 and KCC2, are expressed differently for the excitatory state of Gamma-Amino Butyric Acid (GABA). The present study explored the effect of bumetanide as a selective NKCC1 inhibitor either alone or in combination with the phenobarbital in the pilocarpine model of epilepsy.

**Methods:**

An animal model of Status Epilepticus (SE) was induced with pilocarpine in Wistar male rats followed by phenobarbital and or bumetanide or saline administration for 45 days after the induction of SE by Intraperitoneal (IP) injection. The rats were monitored, their behavior was recorded, and after 24 hours they were sacrificed to study the expression of NKCC1 and KCC2 using real time PCR.

**Results:**

The data showed that the effects of a combination of bumetanide with phenobarbital on frequency rate and duration of seizure attack were more than those of the phenobarbital alone. In addition, in the bumetanide and combined treatment groups, NKCC1 expression decreased significantly, compared with untreated epileptic animals. A delayed decrement in NKCC1/KCC2 expression ratio after bumetanide application was also observed.

**Conclusion:**

The combination of bumetanide with phenobarbital increases the inhibition of SE and maximizes the potential of GABA signaling pathway, and can be considered as an effective therapeutic strategy in patients with epilepsy.

## Highlights

- NKCC1 expression significantly decreased in bumetanide group and combination treatment group.
- Duration of seizure attacks significantly decreased in all treated groups. Combination therapy was significantly more effective than phenobarbital alone.
- The severity of seizure attacks significantly decreased in combination group.
- Frequency of seizure attacks significantly decreased in all treated groups.
- Combination therapy was significantly more effective than phenobarbital alone.

## Plain Language Summary

Chloride transporters, NKCC1 and KCC2 are expressed differently for the excitatory state of Gamma-Amino Butyric Acid (GABA). This study explored the effect of bumetanide as NKCC1 inhibitor alone or in combination with the phenobarbital in temporal lobe epilepsy. Status Epilepticus (SE) was induced with pilocarpine in Wistar male rats followed by phenobarbital and or bumetanide or saline administration for 45 days. After the induction of SE by Intraperitoneal (IP) injection, we monitored rats’ behavior and recorded them. After 24 hours, they were sacrificed to study the expression of NKCC1 and KCC2 using real time PCR. Effects of a combination of bumetanide with phenobarbital on the frequency and duration of seizure attacks were more than those of the phenobarbital alone. In the bumetanide and combined treatment groups, NKCC1 expression decreased significantly, compared with untreated epileptic animals. The combination of bumetanide with phenobarbital increases the inhibition of SE and maximizes the potential of GABA signaling pathway, so it can be considered as an effective therapeutic strategy in patients with epilepsy.

## 1. Introduction

Temporal Lobe Epilepsy (TLE) is a common type of epilepsy in adults. TLE is characterized by seizures that emerge in the limbic system. TLE is often accompanied by initial precipitant damage including febrile seizure, perinatal hypoxia, head trauma, and infection. The initial damage occasionally qualifies as Status Epilepticus (SE), a life-threatening neurologic disorder accompanied by loss of consciousness. A cryptic and seizure-free period follows the accelerated damage and subsequently leads to recurrent seizures (Furman, 2013).

The mechanisms involved in TLE are somehow unknown; however, some reports indicate the disruption of blood-brain barrier, neurodegeneration, inflammation, changes in expression of diverse receptors and ion channels, and development of neural hyperexcitability. Despite wide variety of studies investigated TLE, the mechanisms and risk factors are still unclear (Kahle & Staley, 2008a; Palma et al., 2006). It is suggested that the GABAergic signaling pathway plays a significant role in the emergence of TLE (Brandt, Nozadze, Heuchert, Rattka, & Loscher, 2010).

Gamma-Amino Butyric Acid (GABA) is an excitatory neurotransmitter with excitatory effects in the early stages of development, which is characterized by increased expression of Na^+^-K^+^-2Cl^−^ Cotransporter (NKCC1), and decreased expression of K^+^-Cl^−^ Cotransporter (KCC2) in the brain. NKCC1 increases the intracellular Cl^−^ concentration, but KCC2 shows the opposite effect. Therefore, in neurons with higher expression of NKCC1 (due to lack of KCC2), the opening of Cl^−^ channel by GABA leads to the excision of Cl^−^ and depolarization of neurons (Mazarati, Shin, & Sankar, 2009).

Different pro-epileptogenic brain insults downregulate KCC2 and upregulate NKCC1, increasing intracellular Cl^−^ and hyperpolarization and causing development of neuronal excitation in some regions of brain (Ben-Ari & Holmes, 2005; Blaesse, Airaksinen, Rivera, & Kaila, 2009; Galanopoulou, 2007; Kohling, 2002; Payne, Rivera, Voipio, & Kaila, 2003).

The TLE pilocarpine model demonstrated that the change in GABA equilibrium potential (EGABA) is limited to the epileptogenesis period and likely harbor an important mechanism associated with the appearance of epilepsy (Pathak et al., 2007). Mazarati et al. (2009) found that NKCC1 pharmacological blockade in the neonatal brain might show an antiepileptic effect.

In the present study, rats were treated with bumetanide as a NKCC1 inhibitor after pilocarpine-induced epilepsy. Bumetanide is a very strong diuretic drug, which selectively blocks NKCC1 in submicromolar concentrations, reducing intracellular chloride concentration (Hannaert, Alvarez-Guerra, Pirot, Nazaret, & Garay, 2002; Payne et al., 2003). Bumetanide have neuro-protective effects in rat models with traumatic brain damage (Lu et al., 2006; Lu et al., 2007) ; however, its exact anti-epileptogenic function in TLE models with Recurrent Seizures (RS) has yet to be identified.

Although GABA had an anticonvulsant effect in the neonatal seizure model (using either bumetanide alone or in combination with phenobarbital), the combination of both drugs was more efficient (Dzhala, Brumback, & Staley, 2008). Cleary, et al. (2013) demonstrated that bumetanide increased phenobarbital efficacy in a rat model of hypoxic neonatal seizures. In the current study, the effect of bumetanide, phenobarbital, and combination of both drugs on the pilocarpine model of TLE in adult rats were separately evaluated.

## 2. Methods

### 2.1. Animals

Adult male Wistar rats weighing 250–270 g were housed in a controlled environment with 12:12 light/dark cycle at 22±1°C for two weeks before initiation of the experiment. Rats had free access to food and water. All animal experiments were performed according to the Declaration of Helsinki and the study protocol was approved by the Ethics Committee of Iran University of Medical Sciences.

### 2.2. Pilocarpine-induced epilepsy

To induce SE, pilocarpine hydrochloride, a muscarinic cholinergic agonist (Sigma; 360 mg/kg) was injected to animals Intraperitoneally (IP). Animals were pretreated with cholinergic antagonist, scopolamine methyl nitrate (Sigma; 1 mg/kg IP) 30 minutes before pilocarpine injection to reduce the peripheral cholinergic effects (Ferhat et al., 2003).

The behavior of the rats was observed for several hours after injection, and scored using the Racine classification (Racine, 1972). Only rats that displayed SE (stages 3–5) for 3–4 hours were selected in the current study. To finish seizures, diazepam (7 mg/kg, IP) was injected to the rats. Animals were hand fed after SE until they could eat and drink. After two weeks from the first spontaneous recurrent limbic seizures, the occurrence of spontaneous seizures was confirmed 6–8 hours a day, randomly.

### 2.3. Drug administration

Forty-five days after induction of SE, rats received IP injections of phenobarbital (15 mg/kg), bumetanide (30 mg/kg), or a vehicle (Co). The phenobarbital was diluted in 0.9% normal saline and the bumetanide was dissolved in NaOH 0.1 M and 0.9% normal saline. In addition to the vehicle group, three treatment groups were tested: Phenobarbital alone, bumetanide alone, and a combination of phenobarbital with bumetanide.

Rats were videotaped and their behavior was reviewed and scored by a study-blind investigator for severity, frequency, and duration of tonic-clonic seizures. Origin 7.5 SR6 (Microcal Software; USA) was used for data acquisition and analyses.

### 2.4. Real-time RT-PCR detection of NKCC1 and KCC2 expression

Rats were sacrificed 24 hours after drug administration and the hippocampal tissue was dissected by cold Phosphate Buffered Saline (PBS). Total RNA was extracted from the tissue using RNX-Plus (CinnaGen, Iran). By measuring the Optical Density (OD) at 260 and 280 nm using a UV/VIS spectrophotometer (Ultrospec 2000; Pharmacia), the quantity of the isolated RNA were determined. The quantity and quality of the isolated RNA were determined by measurement of the optical density at 260 and 280 nm using a UV/VIS spectrophotometer (Ultrospec 2000; Pharmacia) and agarose gel electrophoresis, respectively.

The cDNA was generated from 1 μg of total RNA by reverse transcription using the CycleScript Reverse Transcription system (Bioneer, Korea). The mRNA expression levels of NKCC1, KCC2 and Glyceralde-hyde-3-Phosphate Dehydrogenase (GAPDH) were determined by quantitative RT-PCR using a real-time thermal cycler (Rotor-Gene 6000: QIAGEN: Germany). GAPDH mRNA was used as the internal control.

The PCR reactions were set up in a volume of 10 μL containing 1 μL of cDNA, 5 μL AccuPower 2X Green-Starq PCR Master Mix (Bioneer, Korea) and 10 pM of each forward and reverse specific primer (QIAGEN). The reaction conditions were 95°C for 10 min, followed by 40 cycles at 95°C for 15 s and 60°C for one min. Amplification specificity was checked by verifying a single peak on the melting curves. All samples and controls were normalized against reference gene. No template controls and reverse transcriptase control were included in each PCR run. All assays were carried out three times as independent PCR runs for each cDNA sample. The ^ΔΔ^CT method (Livak & Schmittgen, 2001) was used to quantify the amplification-fold difference between groups; each gene expression was normalized with respect to GAPDH mRNA content.

**Figure 1:**
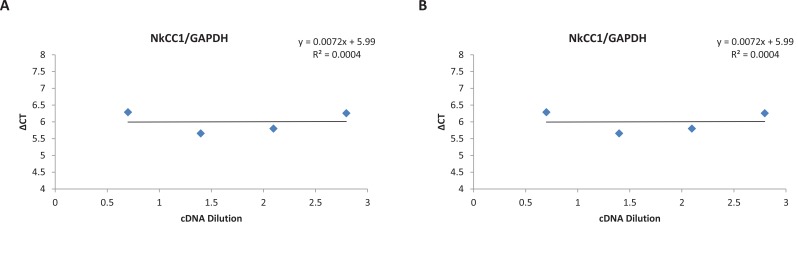
a & b. Validation of ^ΔΔ^CT method The cDNA sample was diluted four times. Serial dilutions were amplified by real-time PCR using reference and target gene primers in triplicate. The average CTs and ^Δ^CT (^Δ^CT _target_–^Δ^CT _reference_) was calculated for each cDNA dilution. The line was fitted using linear regression analysis. A. NKCC1 and GAPDH; B. KCC2 and GAPDH. The slope of the both lines was<0.1; therefore, the ^ΔΔ^CT method can be used to analyze the data (Livak & Schmittgen, 2001).

To validate the use of the ^ΔΔ^CT method, 5-fold serial dilution was performed on a cDNA sample over a 125-fold range. For each dilution sample, PCR was done twice using target and reference genes primers. The average CT of all tests was calculated and the ^Δ^CT of target (NKCC1 and KCC2) and reference (GAPDH) genes was determined. A plot of the log cDNA dilution versus ^Δ^CT(^Δ^CT _target_–^Δ^CT _reference_) was made for each target and reference genes (Figure 1) and the slope of fitted line was determined (Livak & Schmittgen, 2001).

### 2.5. Statistical analysis

All statistical analyses on PCR results were performed with SSPS version 19.0 (SPSS; USA). Oneway Analysis of Variance (ANOVA) with Dunnett post hoc was used to examine significant differences between groups. All results were expressed as Mean±SD.

For monitoring the data, repeated measures and the post hoc Tukey test were used for hour-to-hour and point-to-point analysis of seizure duration and mean seizure score of hour-to-hour severity for the two groups. Two-way ANOVA and post hoc Tukey were used to examine means for seizure attack frequency and duration in 20 hours. All results were presented as Mean±Standard Error of Mean (SEM). For all tests, P<0.05 was considered statistically significant. Origin 7.5 SR6 (Microcal Software, USA) was used for data acquisition and analysis.

## 3. Results

### 3.1. Effects of phenobarbital and/or bumetanide on NKCC1 and KCC2 expression

Phenobarbital (15 mg/kg) and bumetanide (30 mg/kg) were administered alone or in combination during the latent phase of pilocarpine-induced TLE. The animals were sacrificed 24 hours after drug administration and the NKCC1 and KCC2 expression was quantified by RT-PCR.

**Figure 2:**
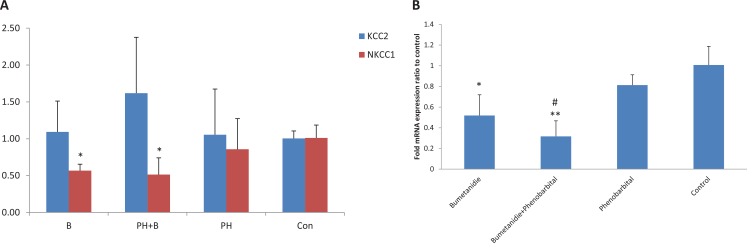
NKCC1 and KCC2 expression A. KCC2 and NKCC1 expression in the hippocampus. NKCC1 and KCC2 expression was quantified by real-time PCR. NKCC1 expression was significantly decreased in bumetanide and combination treatment group compared to untreated epileptic animals. KCC2 expression showed no significant alteration after drug administration. *P<0.05 compared to control group. Results were shown using the Mean±SD. B. NKCC1/KCC2 mRNA expression ratio in the hippocampus. NKCC1/KCC2 ratio was significantly decreased in bumetanide (*P=0.013) and combination groups (**P<0.001) compared to control group and this ratio in combination group was significantly lower (#P=0.003) than that of phenobarbital group. Results were shown using the Mean±SD.

Compared with non-treated epileptic animals, NKCC1 expression decreased significantly in the bumetanide and combined groups. KCC2 expression showed no significant alteration after drug administration (Figure 2a). The hippocampal NKCC1/KCC2 ratio, a good marker of GABA polarity, decreased significantly in the bumetanide (P=0.013) and combined (P<0.001) groups, compared with the control group; this ratio was significantly lower for the combined group (P=0.003) than the phenobarbital group (Figure 2b).

### 3.2. Monitoring of animal for spontaneous recurrent seizures

**Figure 3:**
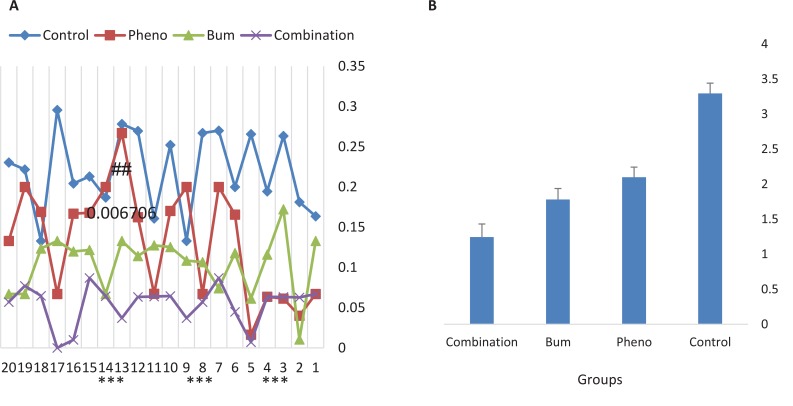
Mean duration of recurrent seizures in 20 hours after drug injection All of the groups were monitored for 20 hours following drug administration. Duration of seizure attacks s were significantly decreased in all treated groups compared to control group. In addition,, combination therapy was significantly more effective than phenobarbital alone. ***P<0.00001 compared to control group; ##P<0.006706 compared to phenobarbital group. Results were shown using the Mean±SD.

**Figure 4:**
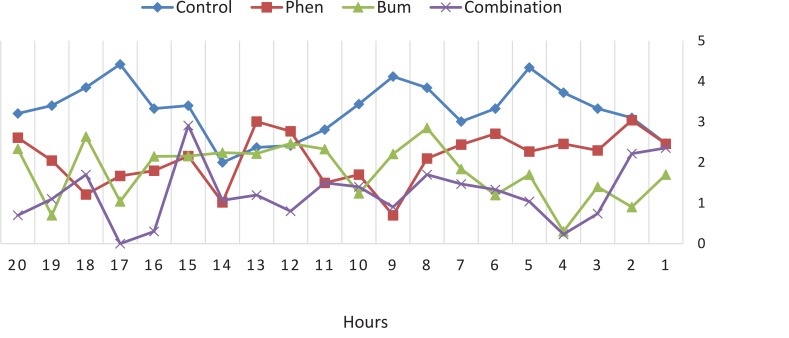
Mean severity of recurrent seizures in 20 hours after drug injection All the groups were monitored for 20 hours after drug administration. The severity of seizure attacks was significantly decreased in combination group. The most reduction in severity was observed 4, 14 and 17 hours after drug injection.

**Figure 5:**
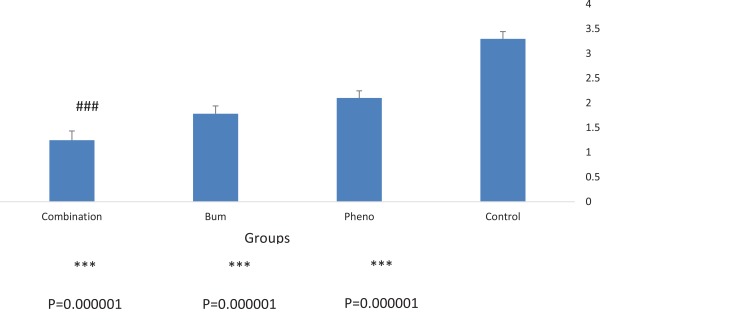
Mean frequency of recurrent seizures in 20 hours after drug injection All the gropus were monitored for 20 hours after drug administration. Frequency of seizure attacks was significantly decreased in all treated groups compared to control group. Also, combination therapy was significantly more effective than phenobarbital alone. ***P<0.000001 compared to control group; ###P<0.000804 compared to phenobarbital group. Results were shown using the Mean±SD.

To compare the efficacy of the drugs, the average severity, frequency, and duration of spontaneous recurrent seizures during the chronic phase of the pilocarpine model of TLE were analyzed. All groups were monitored for 20 hours following drug administration. The data revealed that the effect of combined bumetanide with phenobarbital significantly reduced the duration (Figure 3), severity (Figure 4), and frequency (Figure 5) of seizure attacks.

The highest decrease in severity was observed 4, 14, and 17 hours after drug injection. The frequency and duration of the seizures decreased significantly for all the treatment groups compared with the control group (Figures 3 and 5). The efficacy of combined treatment was significantly higher than the phenobarbital alone.

## 4. Discussion

The present study was performed to investigate whether administration of NKCC1 inhibitor bumetanide alone or with phenobarbital prevents or modifies the development of epilepsy. The results showed that treatment with the combination of bumetanide with phenobarbital decreased the score, frequency, and duration of seizures, and showed higher efficacy compared with phenobarbital alone. The combination of drugs targeting NKCC1 with anticonvulsants increased GABA receptor–mediated conductance exemplify rational anticonvulsant polypharmacy, significantly benefiting some forms of intractable seizures.

Recent studies show the excitatory role of GABAergic signaling in the pathogenesis of TLE that occurs after ischemia in adults (Kahle & Staley, 2008a; Stalłey, 2006). Kahle and Staley (2008b) reported that bumetanide can be effective for the treatment of seizure in adults.

Bumetanide could also be helpful for adult seizures and ischemic encephalopathy by upregulating NKCC1 and decreasing the excitability following such injuries (Kahle & Staley, 2008b). For instance, in cerebral ischemic injury, it is shown that a prolonged increase in [Cl^−^]i leads to hyper-excitability of GABAergic neurons (Galeffi, Sah, Pond, George, & Schwartz-Bloom, 2004; Inglefield & Schwartz-Bloom, 1998). This elevation in [Cl^−^]i, which can be prevented by bumetanide, is associated with increased expression of NKCC1 (Kahle & Staley, 2008b).

For example, neurons in adult TLE accumulate Cl (Cohen, Navarro, Clemenceau, Baulac, & Miles, 2002), probably due to a high ratio of NKCC1 to KCC2 (Kahle & Staley, 2008b). KCC2 can be downregulated by a variety of insults, including brain lesions, spinal cord transections, traumatic insults, and seizures. The evidence of KCC2 internalization after seizure in mice was supported by producing tyrosine phosphorylation of KCC2 and internalization of the cotransporter (Lee, Jurd, & Moss, 2010; Rivera et al., 2004; Wake et al., 2007).

Bumetanide could counteract the depolarizing action of GABA in patients with TLE (Huberfeld et al., 2007; Palma et al., 2006). Cleary et al. (2013) reported that bumetanide in human neonates decreased risk of seizure. During the first postnatal week, NKCC1 expression is at its highest level in cortical neurons of rats, and gradually decreases until next 14 days. Then, it drops to lower limits of adults (Plotkin, Snyder, Hebert, & Delpire, 1997; Wang et al., 2002). Conversely, at birth, the expression of KCC2 is at minimum level in cortical neurons of rats. During the first postnatal week, the expression of KCC2 is low, which increases to adults’ range at the day 14 postnatal (Lu, Karadsheh, & Delpire, 1999).

The higher levels of NKCC1 early in development is accompanied by increasing [Cl^−^]i (Owens & Kriegstein, 2002; Yamada et al., 2004) and excitatory GABA (Ben-Ari, 2002; Dzhala & Staley, 2003; Khazipov et al., 2004). Dzhala et al. (2005) demonstrated similar pattern in the human cortex. This data supports the hypothesis that GABA is excitatory in immature human cortical neurons and neonates, and maybe susceptible to seizures (Kahle & Staley, 2008a).

It therefore seems logical to combine bumetanide (which decreases [Cl^−^]i, and subsequently blocks the excitatory effect of GABA) with phenobarbital (that opens GABAA receptor Cl^−^ channels). The efficiency of bumetanide with phenobarbital was tested for the treatment of recurrent epileptic activity in vitro (Dzhala et al., 2008). Although phenobarbital is not able to diminish the recurrent seizures in 70% of cases, phenobarbital in combination with bumetanide abrogates seizures and significantly reduces the frequency, duration, and power of seizures.

The results of the study indicated the high anticonvulsant efficacy of the combined bumetanide with phenobarbital treatment. In conclusion, combined treatment with bumetanide and phenobarbital after SE increases inhibition and maximizes the anticonvulsant power of the GABA system and can be considered useful for treatment strategy of TLE.

## Ethical Considerations

### Compliance with ethical guidelines

All animal experiments were execute according to the Helsinki declaration and the study was confirmed by the Ethics in Research Committee of Iran University of Medical Sciences.
